# ANGPTL4 Induces TMZ Resistance of Glioblastoma by Promoting Cancer Stemness Enrichment via the EGFR/AKT/4E-BP1 Cascade

**DOI:** 10.3390/ijms20225625

**Published:** 2019-11-11

**Authors:** Yu-Ting Tsai, An-Chih Wu, Wen-Bin Yang, Tzu-Jen Kao, Jian-Ying Chuang, Wen-Chang Chang, Tsung-I. Hsu

**Affiliations:** 1 Graduate Institute of Medical Sciences, College of Medicine, Taipei Medical University, Taipei 110, Taiwan; terry1992@gmail.com (Y.-T.T.); anchinwu@gmail.com (A.-C.W.); geniusbing@gmail.com (W.-B.Y.); 2 Graduate Institute of Neural Regenerative Medicine, College of Medical Science and Technology, Taipei Medical University, Taipei 110, Taiwan; geokao@tmu.edu.tw (T.-J.K.); chuangcy@tmu.edu.tw (J.-Y.C.); 3 Ph.D. Program for Neural Regenerative Medicine, College of Medical Science and Technology, Taipei Medical University and National Health Research Institutes, Taipei 110, Taiwan; 4 TMU Research Center of Neuroscience, Taipei Medical University, Taipei 110, Taiwan; 5 TMU Research Center of Cancer Translational Medicine, Taipei 110, Taiwan

**Keywords:** glioblastoma, angiopoietin-like 4, glioma stem-like cells

## Abstract

Glioblastoma (GBM) is the most aggressive type of brain tumor, with strong invasiveness and a high tolerance to chemotherapy. Despite the current standard treatment combining temozolomide (TMZ) and radiotherapy, glioblastoma can be incurable due to drug resistance. The existence of glioma stem-like cells (GSCs) is considered the major reason for drug resistance. However, the mechanism of GSC enrichment remains unclear. Herein, we found that the expression and secretion of angiopoietin-like 4 protein (ANGPTL4) were clearly increased in GSCs. The overexpression of ANGPTL4 induced GSC enrichment that was characterized by polycomb complex protein BMI-1 and SRY (sex determining region Y)-box 2 (SOX2) expression, resulting in TMZ resistance in GBM. Furthermore, epidermal growth factor receptor (EGFR) phosphorylation induced 4E-BP1 phosphorylation that was required for ANGPTL4-induced GSC enrichment. In particular, ANGPTL4 induced 4E-BP1 phosphorylation by activating phosphoinositide 3-kinase (PI3K)/AKT and extracellular signal–regulated kinase (ERK) cascades for inducing stemness. To elucidate the mechanism contributing to ANGPTL4 upregulation in GSCs, chromatin immunoprecipitation coupled with sequencing (ChIP-Seq) revealed that specificity protein 4 (Sp4) was associated with the promoter region, −979 to −606, and the luciferase reporter assay revealed that Sp4 positively regulated activity of the ANGPTL4 promoter. Moreover, both ANGPTL4 and Sp4 were highly expressed in GBM and resulted in a poor prognosis. Taken together, Sp4-mediated ANGPTL4 upregulation induces GSC enrichment through the EGFR/AKT/4E-BP1 cascade.

## 1. Introduction

Gliomas are the most common type of tumors occurring in the brain, and are classified by the Word Health Organization as grade I–IV, depending on the level of malignancy. In particular, glioblastoma (GBM) is considered a grade IV glioma, with the highest proliferative and invasive capacity among all types of brain tumors [1]. The current standard therapy for GBM includes surgical resection combined with radiation and chemotherapy. Temozolomide (TMZ), an oral alkylating agent, is the most widely used in chemotherapy in GBM treatment [2]. 

However, with the current standard treatment, GBMs remain incurable due to drug resistance. Previous studies indicate that in the heterogeneous tissues of GBM, highly tumorigenic cells called glioblastoma stem cells (GSCs) are thought to cause the recurrence of GBM. Although GSCs represent a small portion of the GBM microenvironment, stemness—i.e., self-renewal and poor differentiation—helps GSCs survive under treatment, leading to the generation of recurrent tumors with a higher tolerance to therapy [3]. Moreover, glioma cells are capable of transforming into GSCs under the treatment of TMZ, resulting in drug resistance [4]. GSCs are characterized by the uncontrolled overexpression of signaling transcription factors, such as the product of *PROM1* gene (CD133), SRY (sex determining region Y)-box 2 (SOX2), and polycomb complex protein BMI-1. SOX2 and BMI-1 inhibit the differentiation signal and keep GSCs in an undifferentiated state, which increases the tolerance of tumors to TMZ-mediated chemotherapy [5,6]. 

Eukaryotic translation initiation factor 4E (eIF4E)-binding protein 1 (4E-BP1) is a key regulator of translation, binding to the eukaryotic initiation factor (eIF) 4E-mRNA cap complex and inhibiting the translation of tumorigenic oncogenes. In most types of cancer, the function of 4E-BP1 is suppressed by protein phosphorylation. Phosphorylation of 4E-BP1 by mammalian target of rapamycin (mTOR), AKT, and extracellular signal–regulated kinase (ERK) prevents 4E-BP1 binding to the eIF-complex, leading to an aberrantly upregulated translational efficiency, which is an important characteristic of tumor growth, metastatic progression, and cancer stem cell enrichment [7,8]. 

Angiopoietin-like 4 protein (ANGPTL4) is part of the angiopoietin (ANG) superfamily which modulates angiogenesis, and is mainly expressed in the liver and adipose tissue [9]. The roles of ANGPTL4 in cancer are still controversial. Several studies indicate that ANGPTL4 promotes cell proliferation, angiogenesis, anoikis resistance, and metastasis in some types of cancer [10,11,12]. However, in melanoma, lung, and colorectal cancer, the induction of ANGPTL4 is reported to inhibit cell growth, angiogenesis, and metastasis [13]. ANGPTL4 expression has been shown to increase with tumor malignancy, and multiple oncogenic signaling was able to positively regulate ANGPTL4 expression [14]. Signal transducer and activator of transcription (STAT) 3 enriches cancer stem cell through upregulating ANGPTL4, and STAT3 inhibitor abrogated STAT3 binding to the ANGPTL4 promoter and exhibited anticancer activity [15]. In addition, epidermal growth factor receptor variant III (EGFRvIII) is able to induce ANGPTL4 expression through the ERK/c-Myc pathway and promotes tumor angiogenesis in malignant gliomas [16]. However, there is a lack of evidence to directly demonstrate effect of ANGPTL4 on cellular sensitivity to chemotherapy. Moreover, the mechanism underlying ANGPTL4-induced drug resistance in GBM remains unknown, prompting our interest to investigate the role of ANGPTL4 in modulating cellular sensitivity to TMZ-mediated chemotherapy and to clarify whether ANGPTL4 participates in GSC enrichment. 

In this study, we found that the induced secretion of ANGPTL4 leads to TMZ resistance and the enrichment of stemness in GBM. In particular, specificity protein (Sp) 4, rather than Sp1, clearly increased ANGPTL4 expression through transcriptional upregulation. Moreover, we found that epidermal growth factor receptor (EGFR) tyrosine kinase and the AKT/4E-BP1 phosphorylation pathway are required by ANGPTL4-induced stemness. Based on this evidence, Sp4-mediated ANGPTL4 expression and secretion induce TMZ resistance through EGFR/AKT/4E-BP1 cascade-mediated stemness enrichment in GBM.

## 2. Results

### 2.1. ANGPTL4 Induces TMZ Resistance in GBM Cells

**Figure 1:**
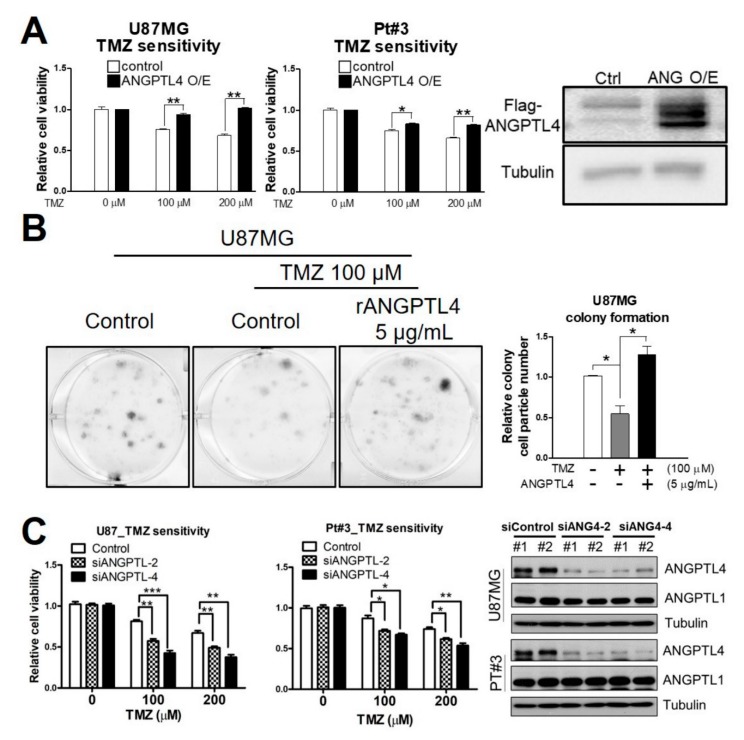
Effect of angiopoietin-like 4 (ANGPTL4) on temozolomide (TMZ) sensitivity in glioblastoma (GBM) (**A**) Cells were transfected with a Flag-ANGPTL4 plasmid for 2 days and then treated with different doses of TMZ for 4 days. Cell viability was measured by an MTT assay. The experiment was performed independently three times, and data were expressed as a relative value ± SEM. (* *p* < 0.05 and ** *p* < 0.01). Western blotting was used to confirm ANGPTL4 overexpression in U87MG cells. (**B**) Cells treated with recombinant ANGPTL4 (rANGPTL4) and TMZ for 5 days were stained with methylene blue. The size of the colony was quantitated using Image J software. The experiment was performed independently three times, and data were expressed as a relative value ± SEM. (* *p* < 0.05). (**C**) After treatment with ANGPTL4 small interfering RNA (siRNA) #2 (siANGPTL4-2) or #4 (siANGPTL4-4) for 3 days, cells were treated with different doses of TMZ for 4 days. Cell viability was measured by an 3-(4,5-Dimethylthiazol-2-yl)-2,5-diphenyltetrazolium bromide (MTT) assay. (** *p* < 0.01 and *** *p* < 0.001). The experiment was performed independently three times. Western blotting was used to confirm ANGPTL4 knockdown. ANGPTL1 expressed was not affected by ANGPTL4 knockdown.

To investigate the role of ANGPTL4 in modulating TMZ sensitivity, U87MG and Pt#3 cell lines either with or without Flag-ANGPTL4 overexpression were treated with TMZ. An 3-(4,5-Dimethylthiazol-2-yl)-2,5-diphenyltetrazolium bromide (MTT) assay revealed that the overexpression of ANGPTL4 reduced the sensitivity of TMZ in GBM cells (Figure 1A and Supplementary Figure S1A). Moreover, human recombinant ANGPTL4 protein (rANGPTL4) treatment obviously attenuated TMZ-induced cytotoxicity in U87MG, as determined using a colony formation assay (Figure 1B). In parallel, to evaluate effect of ANGPTL4 silence on TMZ sensitivity, we confirmed the efficacy of ANGPTL4 small interfering RNA (siRNA) (#1–#4) in Pt#3 cells (Supplementary Figure S2). Knockdown of ANGPTL4 using two independent siRNA (#2 or #4) sensitized U87MG and Pt#3 cells to TMZ (Figure 1C and Supplementary Figure S1B). Therefore, we confirmed that ANGPTL4 participates in inducing the TMZ resistance of GBM.

### 2.2. ANGPTL4 Secretion by GBM Cells Increases GSC Enrichment

**Figure 2:**
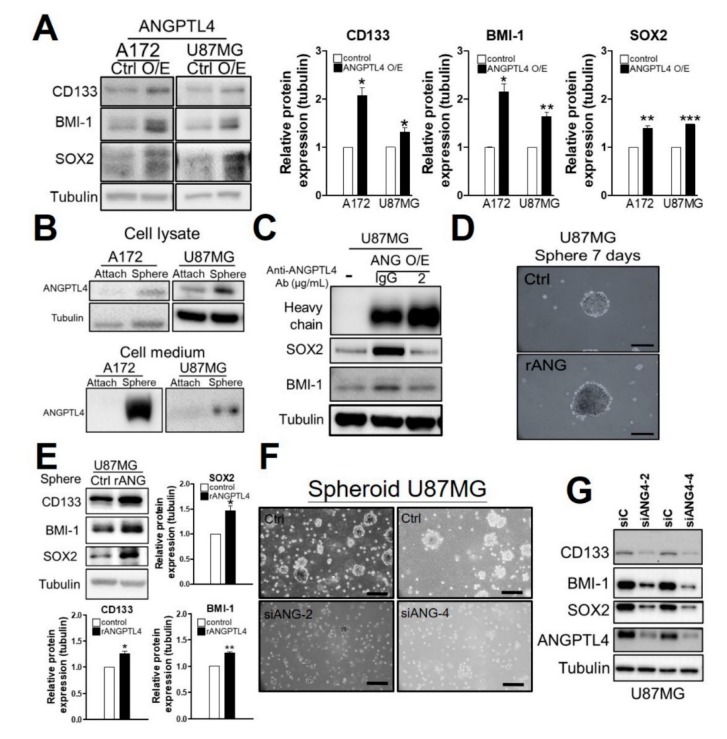
Effect of ANGPTL4 on glioma stem-like cell (GSC) enrichment. (**A**) After ANGPTL4 overexpression for 2 days, the protein expression of stemness markers were presented by Western blotting, including the product of *PROM1* gene (CD133), SRY (sex determining region Y)-box 2 (SOX2), and polycomb complex protein BMI-1. The experiment was performed independently three times, and quantitative results were expressed as the mean ± SEM (* *p* < 0.05, ** *p* < 0.01, and *** *p* < 0.001). (**B**) A comparison of ANGPTL4 production in attached and spheroid cells (GSC-like cells). The protein expression of ANGPTL4 in cell lysate and cultured medium was validated by Western blotting. The cultured medium of cells was collected and concentrated for Western blotting. (**C**) U87MG cells were transfected with a Flag-ANGPTL4 plasmid for 2 days and then treated with the ANGPTL4-neutralizing antibody for an additional 1 day. The protein expression of stemness markers was presented by Western blotting. (**D**) U87MG cells were induced to spheroid formation for 7 days in the absence or presence of rANGPTL4 (5 μg/mL). The scale bar was 0.2 mm. (**E**) After 7 days, cell lysates of spheroid GSCs were collected for Western blotting. The experiment was performed independently three times, and quantitative results were expressed as the mean ± SEM (* *p* < 0.05). (**F**) After transfection with ANGPTL4 siRNA #2 or #4 for 3 days, U87MG cells were induced to spheroid formation for 7 days (the scale bar was 0.5 mm.) and (*G*) the protein expression of stemness markers was validated by Western blotting.

Based on the evidence that GSCs play an important role in the TMZ resistance of GBM [2], we attempted to investigate whether ANGPTL4 is involved in the enrichment of GSCs. As shown in Figure 2A, the overexpression of ANGPTL4 and rANGPTL4 treatment increased the protein expression of stemness markers, including CD133, SOX2, and BMI-1 (Figure 2A and Supplementary Figure S3A,B). Additionally, ANGPTL4 expression was increased in spheroid GSCs compared with adhered GBM cells (Figure 2B top). Given that ANGPTL4 is a secreted protein [11], we postulated that ANGPTL4 is secreted out of cells to enrich GSCs. Therefore, we examined the protein level of ANGPTL4 in the cultured medium of adhered and spheroid cells. We found that the amount of ANGPTL4 protein dramatically increased in spheroid cell-derived medium (Figure 2B bottom), suggesting that GSC-like cells secrete a high level of ANGPTL4. To determine whether secreted ANGPTL4 induces stemness, we evaluated the effect of an ANGPTL4 antibody on stemness. The results showed that the ANGPTL4-neutralizing antibody obviously reduced the protein expression of BMI-1 and SOX2 in the presence of ANGPTL4 overexpression (Figure 2C). Furthermore, we found that rANGPTL4 (5 μg/mL) enhanced spheroid formation and increased the protein expression of CD133, BMI-1, and SOX2 (Figure 2D,E). By contrast, both spheroid formation and stemness markers were reduced by ANGPTL4 knockdown (Figure 2F,G), suggesting that ANGPTL4 secretion participates in GSC enrichment.

### 2.3. ANGPTL4 Regulates Stemness through the EGFR/AKT/4E-BP1 Cascade

**Figure 3:**
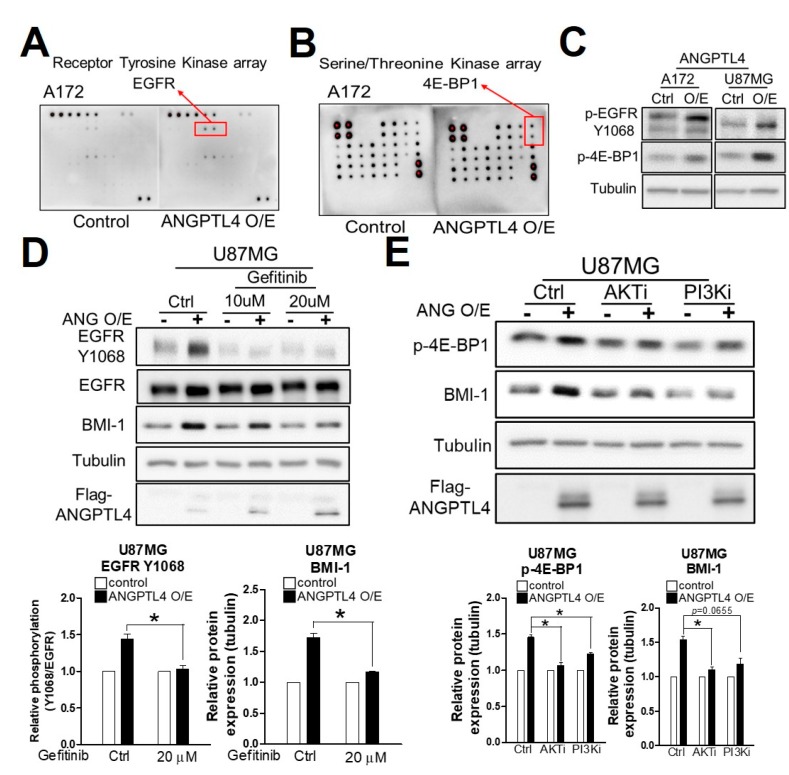
ANGPTL4-regulated phosphorylation cascade for GSC enrichment. (**A**) The putative target receptor of ANGPTL4 was detected by Human receptor tyrosine kinase (RTK) Phosphorylation Antibody Array C1. (**B**) The putative phosphorylation signaling pathway of ANGPTL4 was detected by Human and Mouse AKT Pathway Phosphorylation Array C1. (**C**) Epidermal growth factor receptor (EGFR) phosphorylation (Y1068) and Eukaryotic translation initiation factor 4E (eIF4E)-binding protein 1 (4E-BP1; Thr37/46) phosphorylation in A172 and U87MG were validated by Western blotting. (**D**) After ANGPTL4 overexpression for 24 h, cells were treated with gefitinib for an additional 24 h. The protein expression was validated by Western blotting. The experiment was performed independently 3 times, and quantitative results were expressed as the mean ± SEM (* *p* < 0.05). (**E**) After ANGPTL4 overexpression for 24 h, cells were treated with the AKT or PI3K inhibitor for another 24 h. The protein expressions were validated by Western blotting. The experiment was performed independently three times, and quantitative results were expressed as the mean ± SEM (* *p* < 0.05).

To investigate the signaling pathway regulated by ANGPTL4 for enriching GSCs, we analyzed whether ANGPTL4 induces the phosphorylation of receptor tyrosine kinases (RTKs) and serine/threonine kinases (Supplementary Table S1). Compared with the control, we found that the phosphorylation of EGFR was increased by ANGPTL4 overexpression (Figure 3A) in parallel with an increase in 4E-BP1 phosphorylation (Figure 3B). Furthermore, we confirmed that ANGPTL4 induces extensive EGFR phosphorylation at the Y1068 residue and 4E-BP1 phosphorylation at Thr37/46 residues (Figure 3C and Supplementary Figure S4A–B). Moreover, gefitinib, an inhibitor of EGFR, clearly abolished ANGPTL4-induced BMI-1 expression (Figure 3D and supplementary Figure S4C), suggesting that EGFR phosphorylation is involved in ANGPTL4-induced GSC enrichment. To elucidate which kinase is responsible for 4E-BP1 phosphorylation, we evaluated the effects of inhibitors targeting PI3K, AKT, and ERK—all of which were reported to activate 4E-BP1—on ANGPTL4-induced stemness [7]. As shown in Figure 3E and Supplementary Figure S4D,E, PI3K, AKT, and ERK inhibitors significantly blocked ANGPTL4-induced 4E-BP1 phosphorylation and BMI-1 expression, suggesting that PI3K/AKT and ERK pathways are involved in ANGPTL4-induced GSC enrichment.

### 2.4. Sp4 Increases ANGPTL4 Expression Leading to Stemness Development

**Figure 4:**
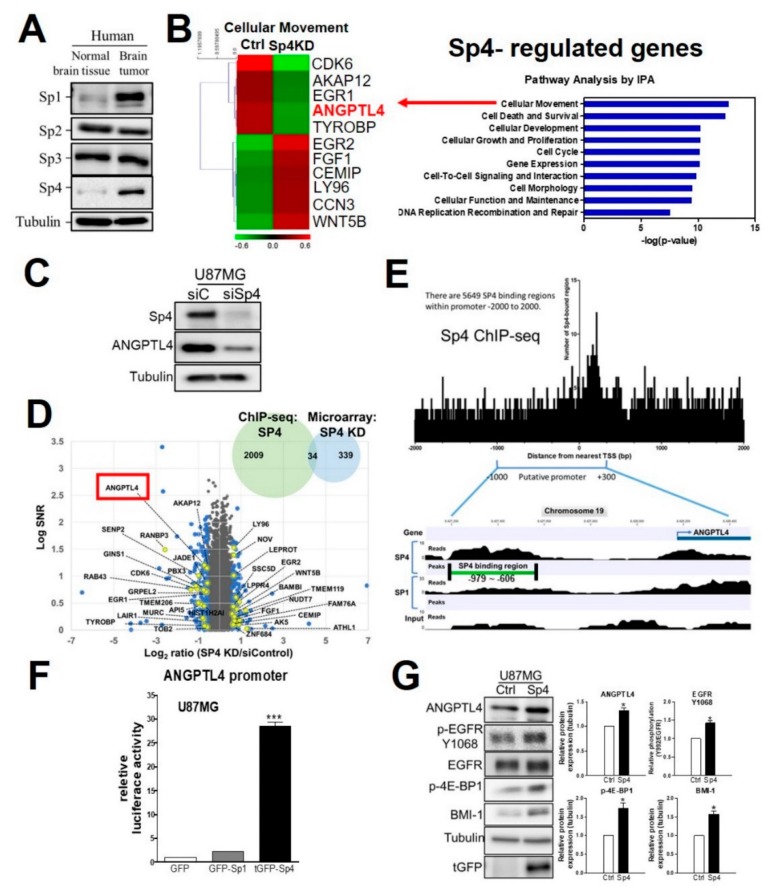
Effect of specificity protein (Sp)4 on ANGPTL4 and GBM. (**A**) The protein expression of Sp1, 2, 3, and 4 in normal brain tissue and a brain tumor was validated by Western blotting. Normal brain tissue lysate: GTX27918 (Genetex); Brain tumor tissue: SF268 (Human Glioblastoma, Origene). (**B**) Left panel: heatmap of Sp4-regulated cell movement-related genes. Right panel: The results of microarray analysis after Ingenuity Pathway Analysis (IPA)-mediated function annotation. (**C**) After Sp4 knockdown for 3 days, the protein expression of ANGPTL4 and Sp4 was validated by Western blotting. (**D**) Sp4-regulated genes in which Sp4 associates with the promoter region revealed by chromatin immunoprecipitation coupled with sequencing (ChIP-Seq). (**E**) The defined promoter region of ANGPTL4 and the binding regions of Sp4 determined by ChIP-seq. (**F**) After transfection with the indicated plasmid and pGL2-ANGPTL4 promoter for 2 days, the promoter activity of ANGPTL4 were measured by a reporter assay. Data were expressed as the relative luciferase activity mean ± SEM. (*** *p* < 0.001). (**G**) After Sp4 overexpression for 48 h, the protein expression was validated by Western blotting. The experiments were performed three times independently, and quantitative results were expressed as the mean ± SEM (* *p* < 0.05).

Among the members of the specificity protein (Sp) family, both Sp1 and Sp4 were obviously increased in brain tumor compared with normal brain (Figure 4A). Microarray analysis showed that Sp4 knockdown decreases ANGPTL4 expression validated by Western blotting in U87MG, and that ANGPTL4 is annotated as being part of the cellular movement group that includes A-kinase anchoring protein 12 (AKAP12), ANGPTL4, cyclin-dependent kinase 6 (CDK6), cell migration-inducing hyaluronidase 1 (CEMIP), early growth response protein 1 (EGR1), early growth response protein 2 (EGR2), fibroblast growth factor 1 (FGF1), lymphocyte antigen 96 (LY96), cellular communication network factor 3 (CCN3), TYRO protein tyrosine kinase binding protein (TYROBP), and Wnt family member 5B (WNT5b) (Figure 4B–C). After a combination of microarray analysis and chromatin immunoprecipitation coupled with sequencing (ChIP-Seq), ANGPTL4 was included in a group of 34 Sp4-regulated genes based on Sp4-regulated expression and the binding affinity of Sp4 (Figure 4D). Furthermore, ChIP-seq provided a putative binding region, from −979 to −606, and the recognized binding sequences for Sp4 on the ANGPTL4 promoter in which Sp4, not Sp1, positively regulated transcriptional activity through binding (Figure 4E and Supplementary Figure S5). In addition, we identified multiple putative Sp1-binding sequences on the ANGPTL4 promoter as shown in Supplementary Table S2. A reporter assay revealed that Sp4, and not Sp1, overexpression significantly increased the promoter activity of ANGPTL4 (Figure 4F). Moreover, we confirmed that Sp4 significantly increased the protein expression of ANGPTL4, phosphor-EGFR, phosphor-4E-BP1, and BMI-1 (Figure 4G), suggesting that Sp1-induced ANGPTL4 induces stemness through the EGFR/4E-BP1 axis. 

### 2.5. High Expression of Sp4 and ANGPTL4 Correlate with a Poor Prognosis of GBM

**Figure 5:**
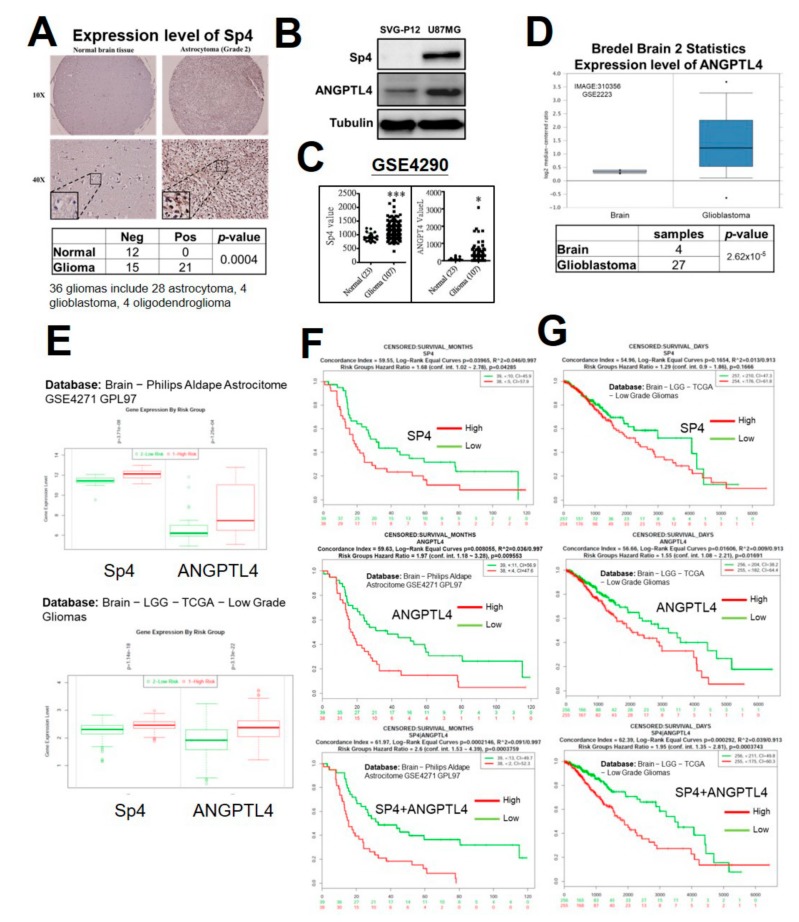
Effect of Sp4 and ANGPTL4 in the clinical relevance of GBM. (**A**) The protein expression of Sp4 and ANGPTL4 in normal brain tissue and glioma was evaluated by immunohistochemical (IHC) staining. (**B**) The protein expression of Sp4 in human fetal glial cells (SVG-P12) and GBM (U87MG) was validated by Western blotting **(C**) The mRNA expression of Sp4 and ANGPTL4 in normal brain tissue and glioma was analyzed using the Gene Expression Omnibus (GEO) dataset (GSE 4290). (**D**) The mRNA expression of ANGPTL4 in normal brain tissue and GBM was analyzed using the Oncomine database (GSE2223). (**E**) The association of Sp4 and ANGPTL4 expression with the risk of glioma. (**F,G**) The correlations of Sp4 and ANGPTL4 with the survival of GBM patients was analyzed using the SurvExpress database (F: brain−Philips Aldape Astrocitome GSE4271 GPL97; G: brain−LGG −TCGA−low grade gliomas).

**Figure 6:**
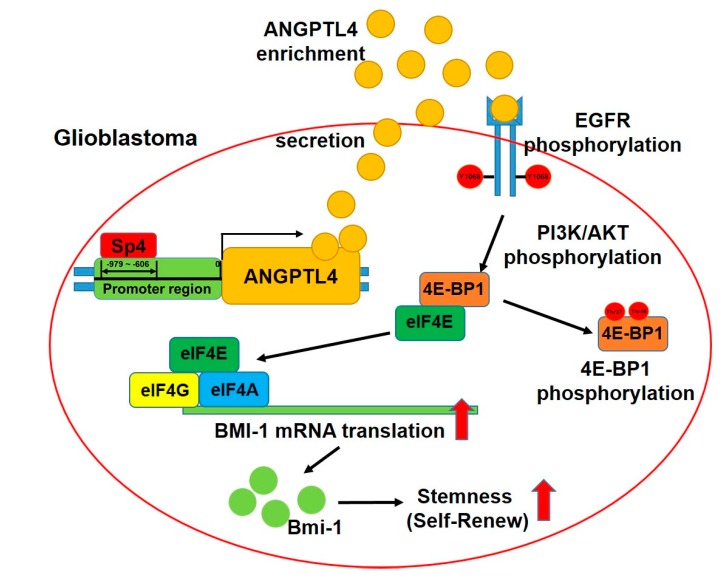
Sp4-mediated ANGPTL4 production induces GSC enrichment through the EGFR/AKT/4E-BP1 cascade, leading to TMZ resistance in GBM. Sp4 induces the expression of ANGPTL4 through binding to the putative promoter region of ANGPTL4, −979 to −606. The secretion of ANGPTL4 induces stemness development by inducing the phosphorylation of EGFR, AKT, and 4E-BP1, leading to the activation of eukaryotic translation initiation factor 4 (eIF4)-mediated cap-dependent translation. The activation of the EGFR/AKT/4E-BP1 cascade by ANGPTL4 results in the upregulation of stemness markers, such as BMI-1, followed by the acquired TMZ resistance.

In contrast to ANGPTL4, which was extensively studied in various types of cancers, Sp4 expression in tumor specimens has never been evaluated. To understand whether Sp4 is overexpressed in GBM, we estimated the level of Sp4 in the tissue array including brain tumor and normal brain tissues using immunohistochemical staining (Figure 5A). In 12 normal brain tissue samples, Sp4 expression was negative; among 36 malignant glioma specimens including 28 astrocytomas, 4 glioblastomas and 4 oligodendrogliomas, Sp4 was positive in 21 specimens (58.33%), indicating that Sp4 expression is significantly increased in glioma (Figure 5A and Supplementary Table S3). As shown in Figure 5B, compared with glial cells, SVG-P12, both of Sp4 and ANGPTL4 were obviously increased in the GBM cell line, U87MG, as determined by Western blotting. To dissect the clinical relevance of Sp4 and ANGPTL4 in GBM, Oncomine and SurvExpress were employed for differential expression and prognosis, respectively. As shown in Figure 5C–D, Sp4 and ANGPTL4 were significantly increased in GBM tissue. Furthermore, high expression of Sp4 or ANGPTL4 significantly associated with high risk of glioma (Figure 5E), and contributed to poor survival in GBM patients (Upper and middle panels, Figure 5F–G). Importantly, high levels of both Sp4 and ANGPTL4 significantly correlated with poor prognosis (lower panel, Figure 5E–F), suggesting a positive correlation of Sp4 with ANGPTL4 in GBM patients. Based on our findings, Sp4-regulated ANGPTL4 production promotes the malignancy of GBM, including the acquirement of drug resistance, by inducing cancer stem cell enrichment (Figure 6).

## 3. Discussion

The enrichment of GSCs, which strongly drives tumors toward drug resistance, remains an obstacle in cancer therapy. Understanding the mechanism underlying GSC development will improve the discovery of novel drugs to overcome drug resistance. Herein, we found that GBM-secreted ANGPTL4 induces TMZ resistance by enhancing GSC enrichment characterized by BMI-1 and SOX2 expression. In particular, Sp4 is responsible for ANGPTL4 upregulation in TMZ-resistant GBM, leading to activation of the EGFR/PI3K/AKT/ERK cascade, which enhances stemness development. However, the role of ANGPTL4 in cancer remains controversial [14]. In giant cell tumor and head and neck squamous cell carcinoma, ANGPTL4 benefits cancers by inducing cell proliferation, angiogenesis, anoikis resistance, and metastasis [10,11]. By contrast, in melanoma, lung, and colorectal cancer, ANGPTL4 inhibits cell growth and angiogenesis [13]. The mechanism causing this discrepancy is still unknown. 

Since ANGPTL4 was considered an orphan ligand [9], we were interested in investigating the targeted receptor tyrosine kinase and signaling pathway regulated by ANGPTL4 for enriching GSC. ANGPTL4 was reported to interact with integrins β1 and β5 to regulate the migration of keratinocyte [17] and be involved in hypoxia-driven vascular permeability by the ANGPTL4- vβ3 axis [18]. In addition, a previous study showed that the interaction between ANGPTL4 and integrins stimulates anoikis resistance through the PI3K and ERK pathway in several tumors [19], suggesting that the integrin subunit is the candidate that can serve as an ANGPTL4 receptor. In our study, we confirmed that the phosphorylation of EGFR was involved in ANGPTL4-induced GSC enrichment and the phosphorylation of 4E-BP1 is significantly induced by ANGPTL4 overexpression. The requirement of EGFR phosphorylation modulates ANGPTL4-activated signaling, suggesting that EGFR is a receptor for ANGPTL4. This is in parallel with other evidence showing that AKT/PI3K inhibitors block the phosphorylation of 4E-BP1, [20] and this results in sensitizing glioblastoma to chemotherapy [21]. Our results showed that AKT and PI3K inhibitors block the phosphorylation of 4E-BP1, resulting in the abolishment of ANGPTL4-induced stemness. In addition, we showed that ANGPTL4 regulates stemness in GBM through the EGFR/AKT/4E-BP1 cascade, which was supported by another study on hepatocellular carcinoma [22]. These results suggest that ANGPTL4 is an important secreted protein that causes drug resistance through inducing cancer stem cell formation.

Multiple signaling cascades were shown to regulate the expression of ANGPTL4, including TGF-β2- and EGF-induced signaling [10] [11]. However, the mechanism by which it contributes to transcriptional upregulation is still unclear. Herein, ANGPTL4 upregulation in GSC was found to be caused by increased Sp4-mediated transcription. In particular, we identified the putative binding region using ChIP-Seq and confirmed that Sp4 overexpression enhances ANGPTL4 promoter activity. Among the members of the Sp family, both Sp1 and Sp4 were obviously increased in brain tumors, and Sp1 has been shown to promote glioma progression and drug resistance in numerous studies. Interestingly, in contrast to Sp4, Sp1 had no affect ANGPTL4 expression. This is probably caused by the discrepancy in recognized sequences between Sp1 and Sp4 revealed by ChIP-Seq (Supplementary Figure S4). Additionally, there are a lack of potential Sp1-binding sites within the 1000 bp promoter which are recognized by Sp4. In previous studies, Sp4 has been shown to be important in regulating gamma-aminobutyric acid-ergic (GABAergic) neurons, and has been correlated with the occurrence of schizophrenia [23]. Before our study showing that Sp4 is involved in the drug resistance of GBM, there was no known role for Sp4 in brain tumors. Therefore, we conclude that Sp4, and not Sp1, increased ANGPTL4 expression, leading to stemness development and poor prognosis. 

## 4. Materials and Methods 

### 4.1. Cell Culture

Human glioblastoma cell lines U87MG and A172 and the human fetal glial cells SVG-P12 were purchased from American Type Culture Collection (ATCC, Manassas, VA, USA), and Pt#3 was derived from a GBM patient [24]. Cells were cultured in Dulbecco’s Modified Eagle Medium (DMEM, Thermo Fisher Scientific, Waltham, MA, USA) supplemented with 10% fetal bovine serum (Thermo Fisher Scientific) and penicillin/streptomycin (Thermo Fisher Scientific) in a humidified atmosphere containing 5% CO_2_. For spheroid cell culture, cells were cultured in serum-free DMEM/F12 (Thermo Fisher Scientific) supplemented with 100 μM sodium pyruvate, 10 ng/mL epidermal growth factor (EGF), 10 ng/mL fibroblast growth factor (FGF) (Thermo Fisher Scientific), and N-2 supplement (Thermo Fisher Scientific) in the culture dishes pre-coated with poly-2-hydroxyethyl methacrylate (poly-HEMA).

### 4.2. Human Specimens and Primary Glioblastoma Cells Pt#3

The use of human tissues was performed based on approval by the Institute Review Board/Ethics Committee from the office of human research at Taipei Medical University (Taipei, Taiwan), as described previously (No. 201006011 and 201402018) [24,25,26].

### 4.3. Chemicals 

Temozolomide (TMZ, Sigma-Aldrich, St. Louis, MO, USA) was dissolved in dimethyl sulfoxide (DMSO, Sigma-Aldrich) to prepare a stock solution of 100 mM. Human ANGPTL4 protein (Fc Tag) (Sino Biological, Wayne, PA, USA) was dissolved in sterile water to prepare a stock solution of 250 μg/mL. Gefitinib (Abcam, Cambridge, UK), AKT (Abcam), PI3K (Abcam), and ERK inhibitors (Abcam) were dissolved in DMSO.

### 4.4. Transfection and Plasmids

GFP-Sp1, tGFP-Sp4 (Origene, Rockville, MD, USA), and ANGPTL4 (GenScript, Piscataway, NJ, USA) were transfected into U87MG, A172, and Pt#3 with PolyJet^TM^ Reagent (SignaGen Laboratories, Rockville, MD, USA) for overexpression. Sp4 and ANGPTL4 siRNA (Dharmacon, Lafayette, CO, USA) were transfected into U87MG, A172, and Pt#3 with Lipofectamine RNAiMAX Reagent (Thermo Fisher Scientific) for knockdown. The ANGPTL4 promoter was designated as the 1000 bp sequences before the first coding region. Restriction enzymes *Hin*dⅢ and *Xho*I (New England Biolabs, Ipswich, MA, USA) were used in the construction of plasmids through ligation with the pGL2-basic vector. The designated ANGPTL4 promoter region on the NCBI database:

(https://www.ncbi.nlm.nih.gov/nuccore/NC_000019.10?report=fasta&from=8363322&to=8364321). 

Forward primer for PCR: CTGGCTCGAGGATGGAGGTCACACGAAGCC.

Reverse primer for PCR: ACCAAGCTTCCTCTTAGGTAGCCTGGGAG.

### 4.5. Western Blotting

Western blotting was performed according to the protocol presented in a previous publication [26], and primary antibodies are listed in Supplementary Table S4.

### 4.6. MTT Assay

An MTT assay was performed according to the protocol presented in a previous publication [26].

### 4.7. Colony Formation

For the colony formation assay, 1000 U87MG cells were seeded into 6-cm dishes. Cells were incubated for a week before being treated with rANGPTL4 (5 μg/mL) and TMZ (100 μM). Cells were treated with rANGPTL4 for 4 days and cells were treated with TMZ in the presence of rANGPTL4 for an additional 5 days. Cells were stained with 1% methylene blue (Sigma-Aldrich).

### 4.8. Phosphorylation Array

Human RTK Phosphorylation Antibody Array C1 (AAH-PRTK-1-4) and Human AKT Pathway Phosphorylation Array C1(AAH-AKT-1-4) (RayBiotech, Inc., Norcross, GA, USA) were used to detect the signaling cascade activated by ANGPTL4.

### 4.9. Reporter Assay

The pGL2-ANGPTL4 plasmid containing the ANGPTL4 promoter region was transfected into U87MG for a reporter assay. Cells were harvested using 1× Cell Culture Lysis Reagent (Promega, San Luis Obispo, CA, USA). A 10 μL sample was mixed with 10 μL luciferin (Promega). A luminometer (HIDEX) was used to measure the promoter activity of the mixture. 

### 4.10. Chromatin Immunoprecipitation Coupled with Sequencing (ChIP-seq) and Data Analysis

U87MG cells were fixed with 1% formaldehyde for preserving the protein–DNA interactions. The cells were then lysed, and chromatin was harvested and analyzed using the Simple ChIP enzymatic chromatin IP kit (#9003, Cell Signaling Technology). The anti-Sp1 and anti-Sp4 antibody were further used to precipitate the fixed DNA–Sp1 and DNA–SP4 complex, respectively. After removing the chromatin proteins by proteinase K, the Sp1- or Sp4-binding DNA fragments were detected using a NextSeq 500 high throughput sequencing system (Illumina, San Diego, CA, USA) and paired-end sequencing with read lengths of 75 bp. Library construction and sequencing were performed by Welgene Biotech (Taipei, Taiwan). The sequencing reads were trimmed and aligned to the genomic sequence retrieved from the reference human genome (UCSC genome browser human genome version hg19). Model-based analysis of ChIP-seq was employed to record ChIP peaks and identify potential binding sites using the CLC Genomics Workbench version 10.1.1 software (Qiagen Bioinformatics). De novo motif discovery was performed on the 2184 sequences of Sp4 binding (putative promoter region from −1000 to + 300) using MEME-ChIP software with default parameters [27]. The Genome Browser views of the Sp1 or Sp4 binding region were created using CLC software. 

### 4.11. Microarray Analysis

Total RNA was extracted using TRIzol reagent (15596026, Thermo Fisher Scientific) from U87MG cells following control or Sp4 siRNA knockdown for 48 h. Gene expression analysis was performed using a SurePrint G3 Human Gene Expression 8 × 60K Microarray by Welgene Biotech Co., Ltd. (Taipei, Taiwan). The number of genes was identified as being differentially expressed, by at least 1.5-fold, and with a signal-to-noise ratio (SNR) ≧ 1, between the control and Sp4 knockdown. Functional analysis was performed by Ingenuity Pathway Analysis (IPA, Qiagen, Denmark). The volcano plot was generated by plotting the log2 ratio (Sp4 KD/siControl) versus the log SNR values of selected genes. The *p*-value calculation for pathway analysis by Ingenuity Pathway Analysis (IPA) analysis was calculated using a Right-Tailed Fisher’s Exact Test (Figure 4B).

### 4.12. Databases

The Gitlab website was employed for the differential expression of Sp4 between brain tissue and GBM. Oncomine was employed for the differential expression of ANGPTL4 between normal brain tissue and GBM in the Bredel Brain 2 Statistics dataset [28,29]. SurvExpress was employed for evaluating the prognostic value of Sp4 and ANGPTL4 in brain−Philips Aldape Astrocitome GSE4271 GPL97 and brain−LGG−TCGA−low grade gliomas datasets [30,31,32]. 

### 4.13. Immunohistochemistry (IHC)

The human tissue array containing normal brain and glioma tissues was purchased from US Biomax, Inc. (Cat. GL241a and T174b, Rockville, MD, USA). The detail information of each tissue spot was provided in Supplementary Table S3. After antigen retrieval, slides were stained by the antibody targeting Sp4 using VECTASTAIN® ABC AP Kits (Vector Laboratories, Inc., Burlingame, CA, USA).

### 4.14. Statistical Analysis

Data were collected from each experiment, which included control and experimental groups. Data were represented as means ± SEM. A one-tailed unpaired Student’s *t*-test was used to analyze the deference between control and experimental groups by the GraphPad Prism 5 program. A probability of *p* < 0.05 was considered significant in all comparisons.

## 5. Conclusions

Based on our evidence, we confirmed that the synthesis and secretion of ANGPTL4 are upregulated in GSC, and that ANGPTL4 induces drug resistance through enhancing GSC enrichment. In particular, through inducing EGFR phosphorylation, ANGPTL4 induces 4E-BP1 phosphorylation via PI3K/AKT and ERK cascades, leading to the increase in BMI-1 expression. Moreover, we elucidate that Sp4, not Sp1, binds to the promoter region and potently increases promoter activity, resulting in the increase in ANGPTL4 production (Figure 6).

## Supplementary Materials

Supplementary materials can be found at https://www.mdpi.com/1422-0067/20/22/5625/s1.

## Abbreviations

4E-BP1	Eukaryotic translation initiation factor 4E (eIF4E)-binding protein 1
AKAP12	A-kinase anchoring protein 12
ANGPTL4	Angiopoietin-like 4 protein
CCN3	Cellular communication network factor 3
CDK6	Cyclin-dependent kinase 6
CEMIP	Cell migration-inducing hyaluronidase 1
ChIP-seq	Chromatin immunoprecipitation coupled with sequencing
DMEM	Dulbecco’s Modified Eagle Medium
DMSO	Dimethyl sulfoxide
DOAJ	Directory of open access journals
EGF	Epidermal growth factor
EGFR	Epidermal growth factor receptor
EGR1	Early growth response protein 1
EGR2	Early growth response protein 2
FGF	Fibroblast growth factor
FGF1	Fibroblast growth factor 1
ERK	Extracellular signal–regulated kinase
GBM	Glioblastoma
GSCs	Glioma stem-like cells
IHC	Immunohistochemistry
IPA	Ingenuity Pathway Analysis
LD	linear dichroism
LY96	Lymphocyte antigen 96
MEME-ChIP	Motif analysis of large DNA datasets
mTOR	Mammalian target of rapamycin
NCBI	National Center for Biotechnology Information
Poly-HEMA	Poly-2-hydroxyethyl methacrylate
PI3K	Phosphoinositide 3-kinase
rANGPTL4	Human recombinant ANGPTL4 protein
RTK	Receptor Tyrosine Kinase
SNR	Signal-to-noise ratio
Sp	Specificity protein
SOX2	SRY (sex determining region Y)-box 2
TLA	Three letter acronym
TMZ	Temozolomide
TYROBP	TYRO protein tyrosine kinase binding protein
WNT5b	Wnt family member 5B
