# Supplementary material for: ANGPTL4 Induces TMZ Resistance of Glioblastoma by Promoting Cancer Stemness Enrichment via the EGFR/AKT/4E-BP1 Cascade
Source: Int J Mol Sci. 2019 Nov 11;20(22):5625. doi: 10.3390/ijms20225625 (PMC6888274; doi:10.3390/ijms20225625)
Supplement: Supplementary file 1 [file ijms-20-05625-s001.pdf]

Supplemental Materials

ANGPTL4 Induces TMZ Resistance of Glioblastoma through Promoting Cancer Stemness Enrichment via the EGFR/AKT/4E-BP1 Cascade

Yu-Ting Tsai, An-Chih Wu, Wen-Bin Yang, Jian-Ying Chuang, Wen-Chang Chang, Tsung-I Hsu

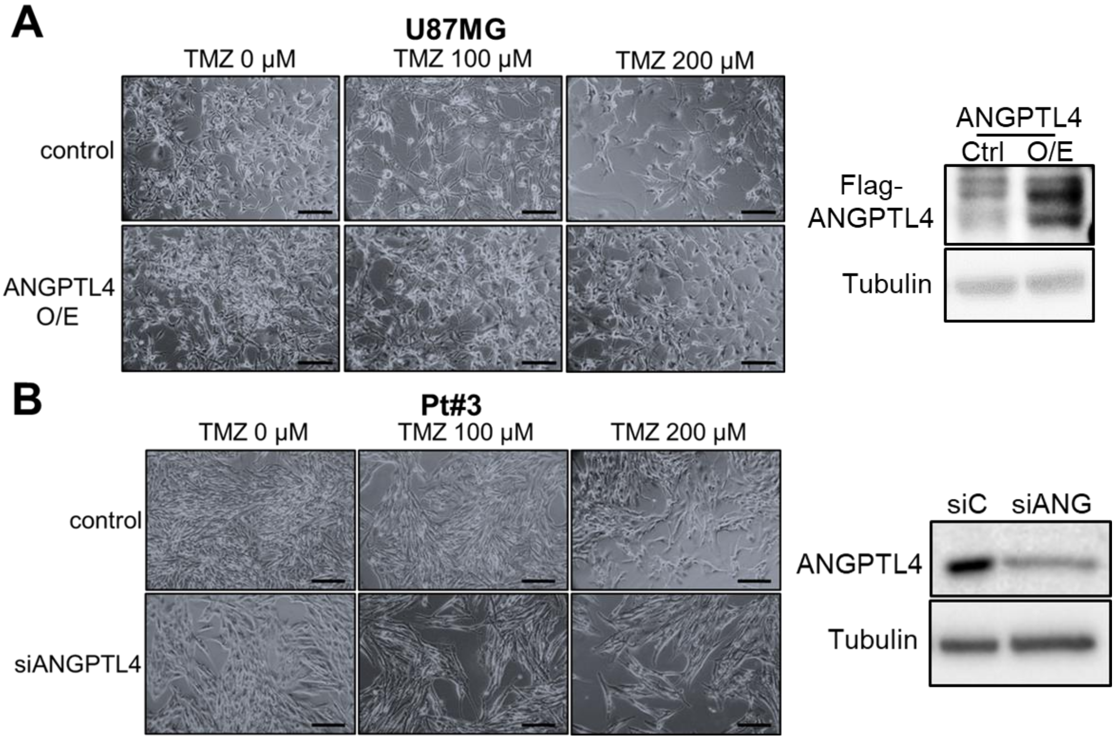

Supplementary Figure S1. Effect of ANGPTL4 on TMZ sensitivity

A. After transfection with Flag-ANGPTL4 plasmid for 2 days, U87MG cells were treated with different doses of TMZ for 4 days. B. After treatment with the siRNA targeting ANGPTL4 for 3 days, Pt#3 cells were treated with different doses of TMZ for 4 days. Left panel: cell morphology was photographed under the microscope. The scale bar was 0.2 mm. Right panel: after 6 (overexpression) or 7 (knockdown) days, cells were harvested, protein lysates were subjected to Western blotting analysis.

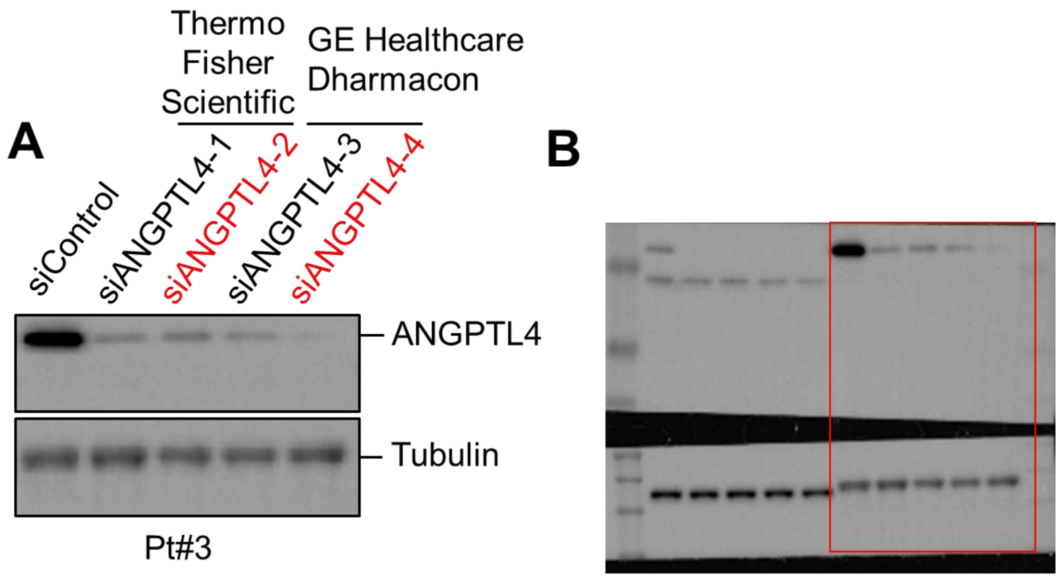

Supplementary Figure S2. Effect of siRNA-targeting ANGPTL4 on ANGPTL4 expression

A. After transfection with siRNA for 3 days, Pt#3 cells were harvested and proteins were collected for western blotting analysis. siANGPTL4-2 and -4 (red) were used in further experiments. B. Original images of Western blotting. Signal marked by red box is used in panel A.

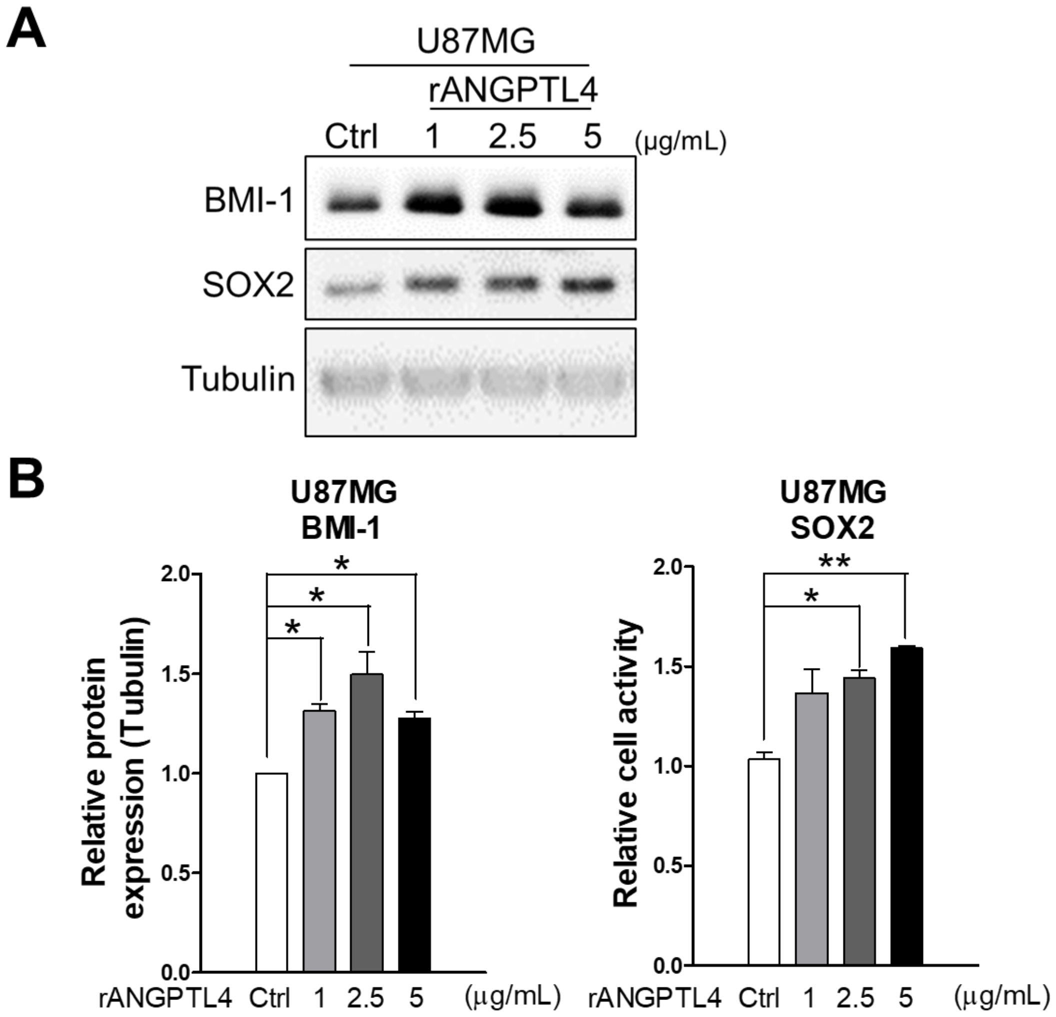

Supplementary Figure S3. Effect of ANGPTL4 on GSC enrichment.

A. After treatment with different doses of rANGPTL4 for 4 days, adhered U87MG cells were harvested for protein preparation followed by western blotting. B. The experiment was performed independently 3 times, and quantitative results were expressed as mean ± SEM (*p<0.05 and **p<0.01).

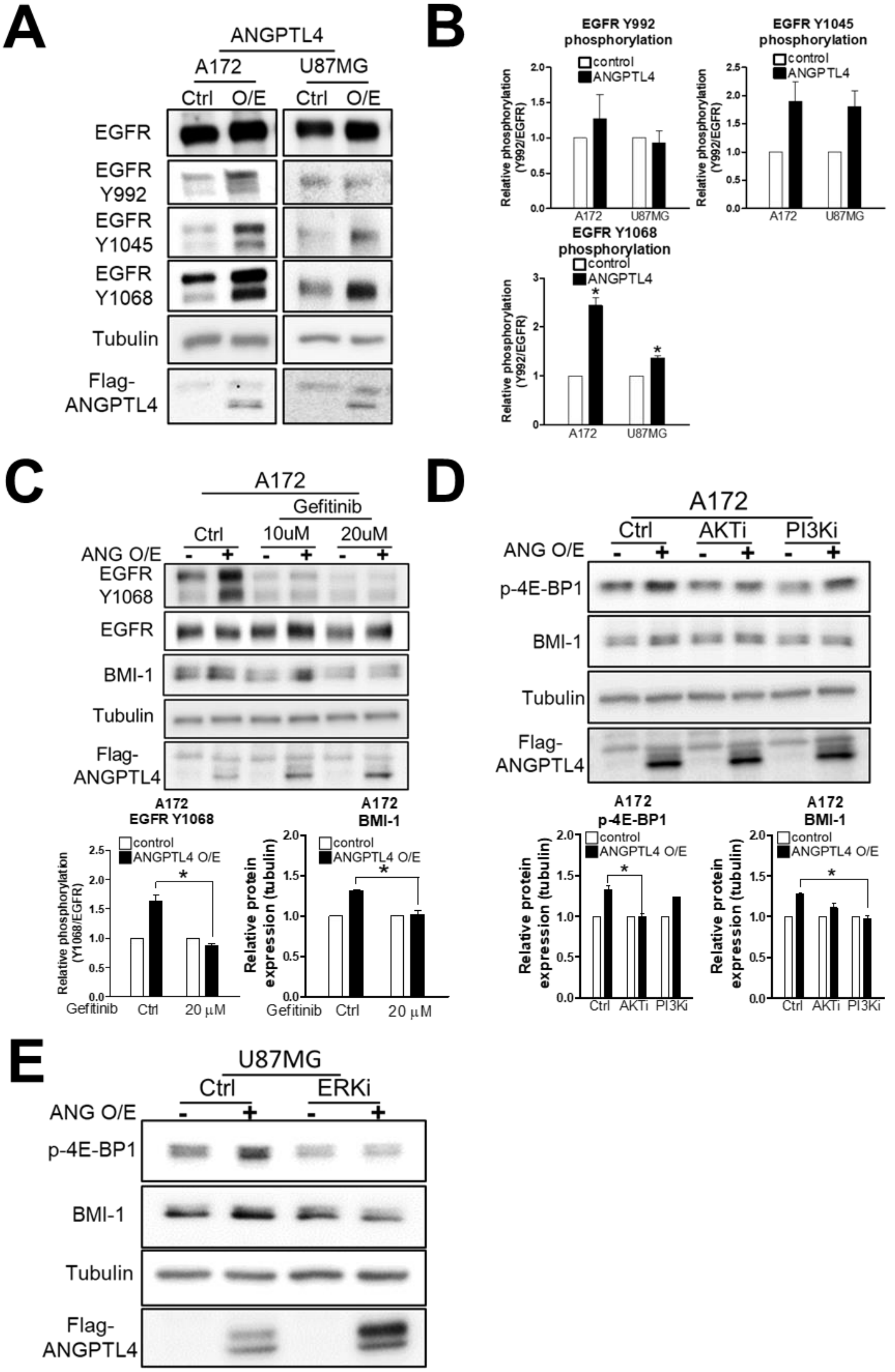

Supplementary Figure S4. ANGPTL4 increases Bmi-1 expression through the EGFR/AKT/4E-BP1 cascade

A. After ANGPTL4 overexpression for 2 days, cell lysates were analyzed for EGFR phosphorylation at Y992, Y1045 and Y1068 residues using western blotting. B. The experiment was performed independently 3 times, and quantitative results were expressed as mean ± SEM (*$p<0.05$). C. After ANGPTL4 overexpression for 24 h, A172 cells were treated with gefitinib (C), AKT (D), PI3K (D) or ERK (E) inhibitor for the additional 24 h. The protein expression was validated by Western blotting. The experiment was performed independently 3 times, and quantitative results were expressed as mean ± SEM (*$p<0.05$).

A

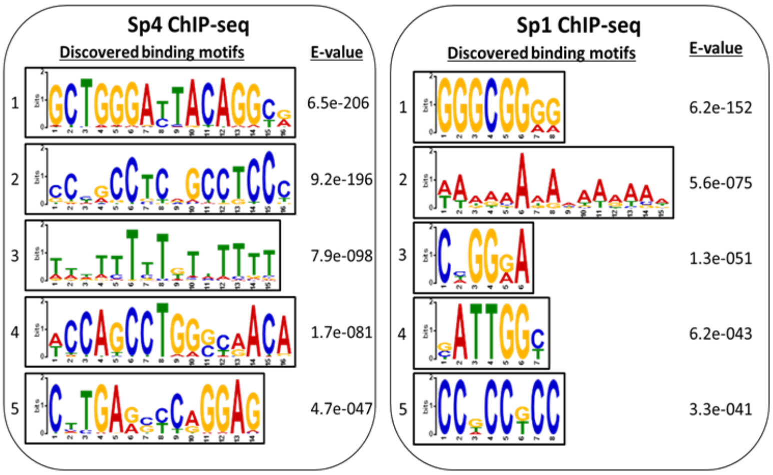

Supplementary Figure S5. Sp1- and Sp4-binding sequences revealed by ChIP-Seq. The top 5 discovered binding motifs of Sp4 and Sp1.

Supplementary Table S1. The list of signaling kinases in phosphorylation kinase array

A. Array map of Human RTK Phosphorylation Antibody Array C1 (related to Figure 3A)

	A	B	C	D	E	F	G	H	I	J	K	L
1	POS1	POS1	POS2	POS2	POS3	POS3	ABL1	ABL1	ACK1	ACK1	ALK	ALK
2	NEG	NEG	NEG	NEG	Axl	Axl	Blk	Blk	BMX	BMX	Btk	Btk
3	Csk	Csk	Dtk	Dtk	EGFR	EGFR	EphA1	EphA1	EphA2	EphA2	EphA3	EphA3
4	EphA4	EphA4	EphA5	EphA5	EphA6	EphA6	EphA7	EphA7	EphA8	EphA8	EphB1	EphB1
5	EphB2	EphB2	EphB3	EphB3	EphB4	EphB4	EphB6	EphB6	ErbB2	ErbB2	ErbB3	ErbB3
6	ErbB4	ErbB4	FAK	FAK	FER	FER	FGFR1	FGFR1	FGFR2	FGFR2	FGFR2 (α isoform)	FGFR2 (α isoform)
7	Fgr	Fgr	FRK	FRK	Fyn	Fyn	Hck	Hck	HGFR	HGFR	IGF-IR	IGF-IR
8	Insulin R	Insulin R	Itk	Itk	JAK1	JAK1	JAK2	JAK2	JAK3	JAK3	LCK	LCK
9	LTK	LTK	Lyn	Lyn	MATK	MATK	M-CSFR	M-CSFR	MUSK	MUSK	NGFR	NGFR
10	PDGFR-α	PDGFR-α	PDGFR-ß	PDGFR-ß	PYK2	PYK2	RET	RET	ROR1	ROR1	ROR2	ROR2
11	ROS	ROS	RYK	RYK	SCFR	SCFR	SRMS	SRMS	SYK	SYK	Tec	Tec
12	Tie-1	Tie-1	Tie-2	Tie-2	TNK1	TNK1	TRKB	TRKB	TXK	TXK	NEG	NEG
13	Tyk2	Tyk2	TYRO10	TYRO10	VEGFR2	VEGFR2	VEGFR3	VEGFR3	ZAP70	ZAP70	POS4	POS4

https://www.raybiotech.com/c-series-human-rtk-phosphorylation-array-1-2/

B. Array map of Human and Mouse AKT Pathway Phosphorylation Array C1 (Related to Figure 3B)

Each antibody is spotted in duplicate vertically

1<br>2	POS	POS	NEG	NEG	Akt (P-Ser473)	AMPKa (P-Thr172)	BAD (P-Ser112)	4E-BP1 (P-Thr36)
3<br>4	ERK1 (P-T202/Y204)<br>ERK2 (P-Y185/Y187)	GSK3a (P-Ser21)	GSK3b (P-Ser9)	mTOR (P-Ser2448)	p27 (P-Thr198)	P53 (P-Ser15)	P70S6K (P-Thr421/Ser424)	PDK1 (P-Ser241)
5<br>6	PRAS40 (P-Thr246)	PTEN (P-Ser380)	Raf-1 (Ser301)	RPS6 (P-Ser235/236)	RSK1 (P-Ser380)	RSK2 (P-Ser386)	NEG	POS

https://www.raybiotech.com/human-mouse-akt-array/

Supplementary Table S2. Sp4-binding sequences on the ANGPTL4 promoter revealed by ChIP-Seq

Sp4 binding sequence	Putative sites on ANGPTL4 promoter region
GCTGGGACTACAGGCG	-409/-394
GCAACCTCCACCTCCC	-462/-447
ACTGCCTCAGCCTCCC	-430/-415
TTTTTTTTGTATTTT	-371/-357
TCCAGCCTGGGCGACA	-504/-489
ACCAGCCCGGCCAACA	-334/-319
CTTGAACCCGGGAG	-451/-438

Supplementary Table S3. The characteristics of tissues included in the tissue array

T174b

Mouseover and click individual cores to view high resolution images.

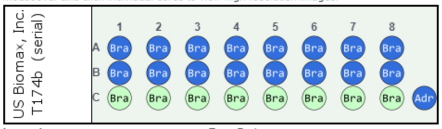

Legend: Bra - Brain

- Malignant tumor, - Normal tissue

Specification Sheet (Sortable), tissue IDs are available in exported Excel files.

Pos.	No.	Age	Sex	Organ/Anatomic Site	Pathology diagnosis	TNM	Grade	Stage	Type	Notes	Image
A1	1	63	M	Brain	Astrocytoma		2		Malignant		
A2	2	63	M	Brain	Astrocytoma		3		Malignant		
A3	3	40	F	Brain	Astrocytoma of right temporal lobe		3		Malignant		
A4	4	40	F	Brain	Astrocytoma of right temporal lobe		3		Malignant		
A5	5	63	M	Brain	Astrocytoma		3		Malignant		
A6	6	63	M	Brain	Astrocytoma		3		Malignant		
A7	7	40	F	Brain	Astrocytoma of right temporal lobe		3		Malignant		
A8	8	40	F	Brain	Astrocytoma of right temporal lobe		3		Malignant		
B1	9	35	M	Brain	Glioblastoma of right frontal lobe		4		Malignant		
B2	10	35	M	Brain	Glioblastoma of right frontal lobe		4		Malignant		
B3	11	35	M	Brain	Anaplastic oligodendroglioma of right frontal lobe		2		Malignant		
B4	12	35	M	Brain	Anaplastic oligodendroglioma (sparse) of right frontal lobe		3		Malignant		
B5	13	35	M	Brain	Glioblastoma of right frontal lobe		4		Malignant		
B6	14	35	M	Brain	Glioblastoma of right frontal lobe		4		Malignant		
B7	15	35	M	Brain	Anaplastic oligodendroglioma of right frontal lobe		2		Malignant		
B8	16	35	M	Brain	Anaplastic oligodendroglioma of right frontal lobe		2		Malignant		
C1	17	28	F	Brain	Cerebrum tissue		-		Normal		
C2	18	28	F	Brain	Cerebrum tissue		-		Normal		
C3	19	50	F	Brain	Cerebrum tissue		-		Normal		
C4	20	50	F	Brain	Cerebrum tissue		-		Normal		
C5	21	28	F	Brain	Cerebrum tissue		-		Normal		
C6	22	28	F	Brain	Cerebrum tissue		-		Normal		
C7	23	50	F	Brain	Cerebrum tissue		-		Normal		
C8	24	50	F	Brain	Cerebrum tissue		-		Normal		
	0	42	M	Adrenal gland	Pheochromocytoma (tissue marker)		-		Malignant	-	

https://www.biomax.us/tissue-arrays/Brain/T174b

GL241a

Mouseover and click individual cores to view high resolution images.

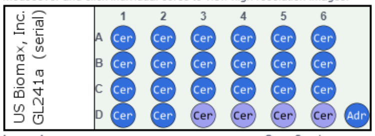

Legend: Cer - Cerebrum

- Malignant tumor, - NAT

Specification Sheet (Sortable), tissue IDs are available in exported Excel files.

Pos.	No.	Age	Sex	Organ/Anatomic Site	Pathology diagnosis	TNM	Grade	Stage	Type	Image
A1	1	33	M	Cerebrum	Astrocytoma		1		malignant	
A2	2	33	M	Cerebrum	Astrocytoma		1		malignant	
A3	3	44	F	Cerebrum	Astrocytoma		1		malignant	
A4	4	44	F	Cerebrum	Astrocytoma		1		malignant	
A5	5	53	M	Cerebrum	Astrocytoma		2		malignant	
A6	6	53	M	Cerebrum	Astrocytoma		2		malignant	
B1	7	34	M	Cerebrum	Astrocytoma		2		malignant	
B2	8	34	M	Cerebrum	Astrocytoma		2		malignant	
B3	9	59	M	Cerebrum	Astrocytoma		2--3		malignant	
B4	10	59	M	Cerebrum	Astrocytoma		2--3		malignant	
B5	11	28	F	Cerebrum	Astrocytoma		1		malignant	
B6	12	28	F	Cerebrum	Astrocytoma		1		malignant	
C1	13	43	M	Cerebrum	Astrocytoma		2		malignant	
C2	14	43	M	Cerebrum	Astrocytoma		2		malignant	
C3	15	35	M	Cerebrum	Astrocytoma		2		malignant	
C4	16	35	M	Cerebrum	Astrocytoma		2		malignant	
C5	17	48	F	Cerebrum	Astrocytoma		3		malignant	
C6	18	48	F	Cerebrum	Astrocytoma		3		malignant	
D1	19	36	M	Cerebrum	Astrocytoma		3		malignant	
D2	20	36	M	Cerebrum	Astrocytoma		3		malignant	
D3	21	44	F	Cerebrum	Cancer adjacent normal brain tissue		-		NAT	
D4	22	44	F	Cerebrum	Cancer adjacent normal brain tissue		-		NAT	
D5	23	28	F	Cerebrum	Cancer adjacent normal brain tissue		-		NAT	
D6	24	28	F	Cerebrum	Cancer adjacent normal brain tissue		-		NAT	
-	0	42	M	Adrenal gland	Pheochromocytoma (tissue marker)		-		Malignant	

https://www.biomax.us/tissue-arrays/Brain/GL241a

Supplementary Table S4. The antibody used in this study

Antibodies	Cat.	resources	Dilution
ANGPTL4	GTX114198	GeneTex, Inc., Irvine, CA, United States	1:1000
BMI-1	Ab126783	Abcam, Cambridge, UK	1:1000
CD133	18470-1-AP	Proteintech Group, Chicago, IL, United States	1:1000
EGFR	4267T	Cell Signaling Technology, Danvers, MA, United States	1:1000
p-EGFR (Y1068)	3777T	Cell Signaling Technology, Danvers, MA, United States	1:1000
Flag-DDK	F1804	Sigma-Aldrich, St. Louis, MO, United States	1:1000
GFP	632592	Takara Clontech Laboratories, Inc., CA, United States	1:5000
tGFP	TA150041	Origene, Rockville, MD, United States	1:1000
Sp1	07-645	MERCK Millipore, Billerica, MA, United States	1:5000
Sp2	Sc-17814	Santa Cruz Biotechnology, Inc., Dallas, TX, United States	1:1000
Sp3	Sc-28305	Santa Cruz Biotechnology, Inc., Dallas, TX, United States	1:1000
Sp4	TA335786	Origene, Rockville, MD, United States	1:1000
SOX2	GTX 101507	GeneTex, Inc., Irvine, CA, United States	1:1000
α-Tubulin	66031-1-lg	Proteintech Group, Chicago, IL, United States	1:10000
p-4E-BP1 (Thr37/46)	2855S	Cell Signaling Technology, Danvers, MA, United States	1:1000
